# Treatment effects on neurometabolite levels in schizophrenia: A meta-analysis dataset of proton magnetic resonance spectroscopy

**DOI:** 10.1016/j.dib.2020.105862

**Published:** 2020-06-16

**Authors:** Manabu Kubota, Sho Moriguchi, Keisuke Takahata, Shinichiro Nakajima, Nobuyuki Horita

**Affiliations:** a Department of Functional Brain Imaging, National Institute of Radiological Sciences, National Institutes for Quantum and Radiological Science and Technology, 4-9-1 Anagawa, Inage-ku, Chiba 263-8555, Japan; b Department of Psychiatry, Kyoto University Graduate School of Medicine, 54 Shogoin-Kawahara-cho, Sakyo-ku, Kyoto 606-8507, Japan; c Research Imaging Centre, Centre for Addiction and Mental Health, Toronto, 250 College Street, Toronto, Ontario M5T1R8, Canada; d Department of Neuropsychiatry, Keio University Graduate School of Medicine, 35 Shinanomachi, Shinjuku-ku, Tokyo 160-8582, Japan; e Yokohama City University Graduate School of Medicine, 3-9 Fukuura, Kanazawa-ku, Yokohama 236-0004, Japan

**Keywords:** MRS, Glutamate, Glutamine, Gamma-aminobutyric acid, *N*-acetylaspartate, Myo-inositol, Antipsychotic, Psychosis

## Abstract

This article describes a dataset for a meta-analysis that aimed to investigate the effects of treatment on the neurometabolite status in patients with schizophrenia (DOI of original article: https://doi.org/10.1016/j.schres.2020.03.069[1]). The data search was performed with MEDLINE, Embase, and PsycINFO. The neurometabolites investigated include glutamate, glutamine, glutamate + glutamine, gamma-aminobutyric acid, *N*-acetylaspartate, and myo-inositol, and the regions of interest (ROIs) include the frontal cortex, temporal cortex, parieto-occipital cortex, thalamus, basal ganglia, and hippocampus. The meta-analysis was conducted with a random-effects model, and the use of the standardized mean difference method between pre- and post-treatment of subjects for neurometabolites in each ROI of three patient groups or more. The dataset covers raw data of 39 patient groups (773 patients with schizophrenia at follow-up) with neurometabolite levels measured by magnetic resonance spectroscopy both before and after treatment. Furthermore, it contains details of clinical characteristics and treatment types for each group. Therefore, the data would be useful for a reinvestigation of treatment effects on the neurometabolite status from diverse points of view, as well as for the development of future treatment strategies for psychiatric diseases.

Specifications table

**Table:** 

Subject	Psychiatry and Mental Health
Specific subject area	Meta-analysis of proton magnetic resonance spectroscopy (^1^H-MRS) data of treatment effects on neurometabolite levels in schizophrenia [1]
Type of data	Table Figure Plot
How data were acquired	We used the following search terms: (MRS OR "magnetic resonance spectroscopy") AND (schizophrenia OR schizoaffective OR psychosis OR "high risk" OR UHR OR ARMS OR prodrom*). Any English-language articles were included, while non-English articles and conference abstracts were excluded.
Data format	Raw Analyzed
Parameters for data collection	From longitudinal and randomized control research, we collected MRS data of both before and after treatment in patients with schizophrenia. MRS data: glutamate (Glu), glutamine (Gln), glutamate + glutamine (Glx), GABA, *N*-acetylaspartate (NAA), myo-inositol (MI)
Description of data collection	We followed the Preferred Reporting Items for Systematic Reviews and Meta-Analysis (PRISMA) statement [2]. The search was performed with MEDLINE, Embase, and PsycINFO.
Data source location	Institution: National Institute of Radiological Sciences, National Institutes for Quantum and Radiological Science and Technology City: Chiba Country: Japan Latitude and longitude for collected data: (35.636045, 140.103724)
Data accessibility	With the article
Related research article	M. Kubota, S. Moriguchi, K. Takahata, S. Nakajima, N. Horita, Treatment effects on neurometabolite levels in schizophrenia: A systematic review and meta-analysis of proton magnetic resonance spectroscopy studies, Schizophr. Res. (in press) https://doi.org/10.1016/j.schres.2020.03.069[1].

## Value of the data

The dataset covers 39 patient groups (773 patients with schizophrenia at follow-up) with neurometabolite data for both before and after treatment, which allows for a reinvestigation of the treatment effects on the neurometabolite status from diverse points of view.
The dataset includes details of the clinical backgrounds and treatment types for each patient group, facilitating the development of future treatment strategies for psychiatric diseases.
The dataset would be useful for conducting a future meta-analysis associated with treatment intervention in various diseases.

## 1 Data description

Table 1 demonstrates scores of Quality Assessment conducted by modified Newcastle – Ottawa Quality Assessment Scale.

**Table 1:** Modified Newcastle-Ottawa Scale.

Study	Selection				Exposure			
	Case definition	Representative-ness	Ascertainment of exposure	Definition of controls	Assessment of outcome	Follow-Up period	Adequacy of Follow-Up	Total
Aoyama (2011)	2	2	2	2	1	2	2	13
Bustillo (2008)	2	2	2	1	1	2	0	10
Bustillo (2010)	2	2	2	1	1	2	0	10
Conus (2018)	2	1	2	0	2	2	2	11
Dempster (2015)	2	2	2	1	1	2	2	12
Dlabac-de Lange (2017)	2	1	2	0	2	2	2	11
Egerton (2018) (Glostrup)	2	2	2	1	1	2	0	10
Egerton (2018) (London)	2	2	2	1	1	2	0	10
Egerton (2018) (Utrecht)	2	2	2	1	1	2	2	12
Ertugrul (2009)	2	1	2	2	1	2	2	12
Fannon (2003)	2	2	2	1	1	2	0	10
Fannon (2003)	2	2	2	2	1	2	0	11
Fuente-Sandoval (2013)	2	2	2	2	1	2	2	13
Fuente-Sandoval (2017)	2	2	2	2	1	2	2	13
Gan (2014)	2	1	2	2	1	2	2	12
Gan (2017)	2	2	2	2	2	2	2	14
Gan (2017)	2	2	2	2	2	2	2	14
Goff (2002)	2	2	2	0	1	2	0	9
Goto (2012)	2	2	2	0	1	2	2	11
Grosic (2014)	2	2	1	2	1	2	2	12
Grosic (2014)	2	2	2	2	1	2	2	13
Huang (2019)	2	2	1	2	1	2	2	12
Jarskog (2013)	2	2	2	0	2	2	2	12
Kelemen (2013)	2	2	2	2	1	2	2	13
Kraguljac (2019)	1	2	2	2	1	2	0	10
Liemburg (2018)	2	1	2	0	2	2	2	11
Liemburg (2018)	2	1	2	0	2	2	2	11
Marenco (2016)	2	2	2	2	1	0	2	11
McQueen(2018)	2	2	2	0	2	0	0	8
Ota(2015)	2	2	2	0	1	2	2	11
Pae(2004)	2	2	2	2	1	2	0	11
Pajonk(2010)	2	1	2	0	2	2	2	11
Pillinger(2019)	2	1	2	0	1	0	2	8
Premkumar(2010)	2	2	2	0	1	2	0	9
Strzelecki(2015)	2	1	2	0	2	2	2	11
Szulc(2005)	2	2	2	2	1	2	2	13
Szulc(2011)	2	2	2	2	1	2	2	13
Xia(2018)	2	2	1	0	1	2	2	10
Xia(2018)	2	1	2	0	1	2	2	10

**Fig. 1:**
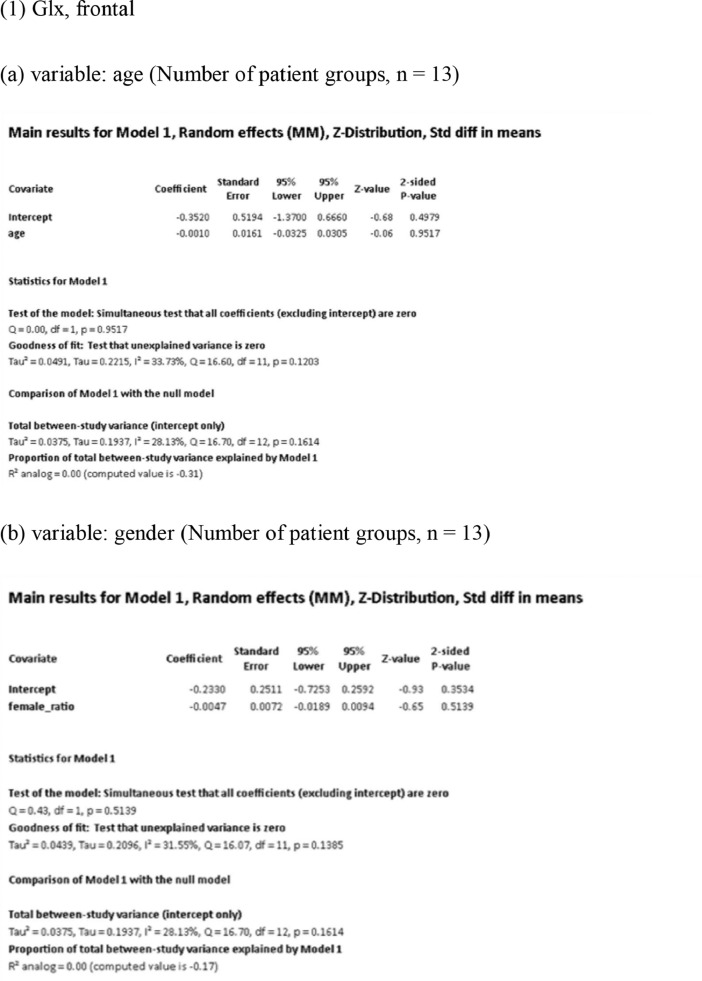

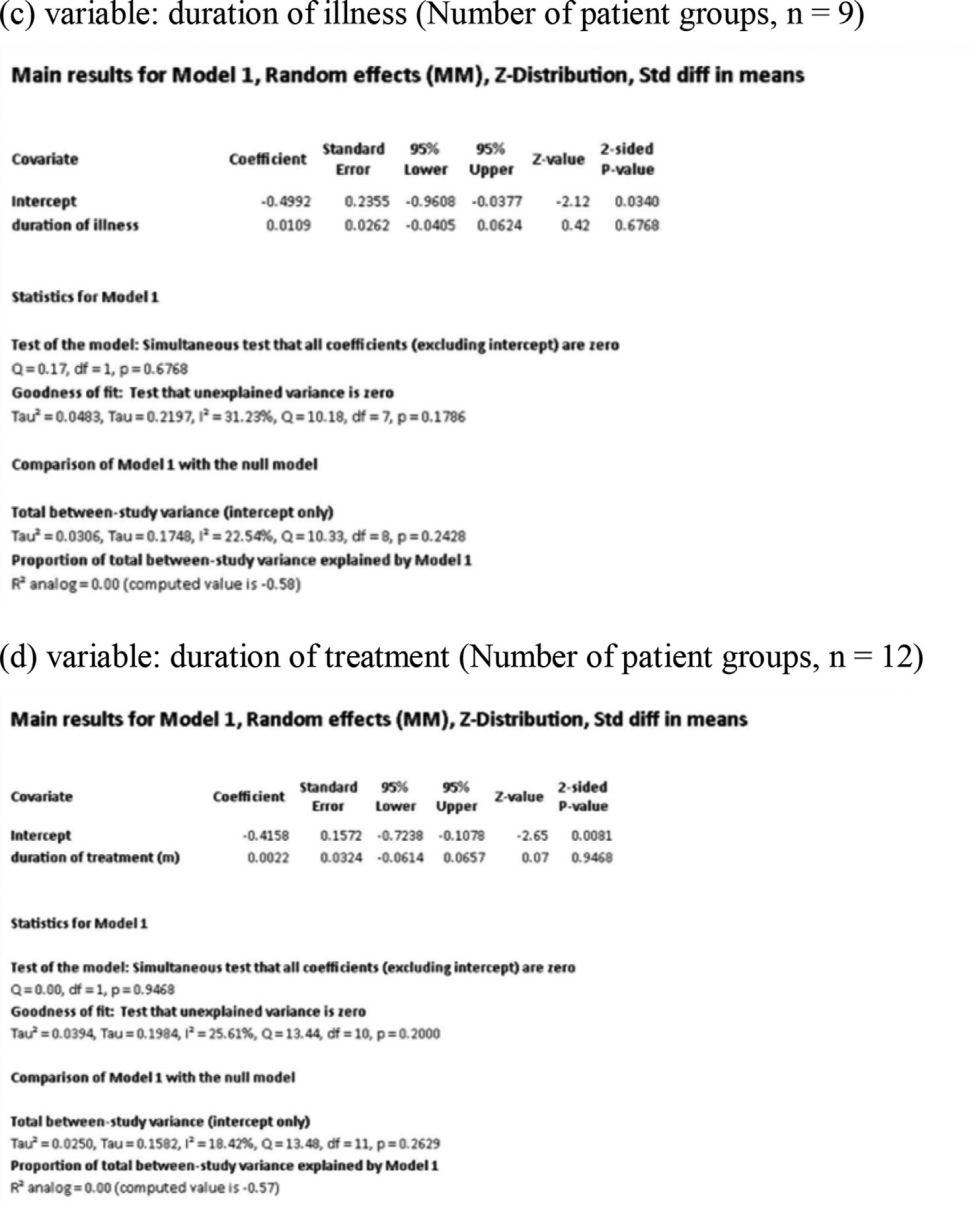

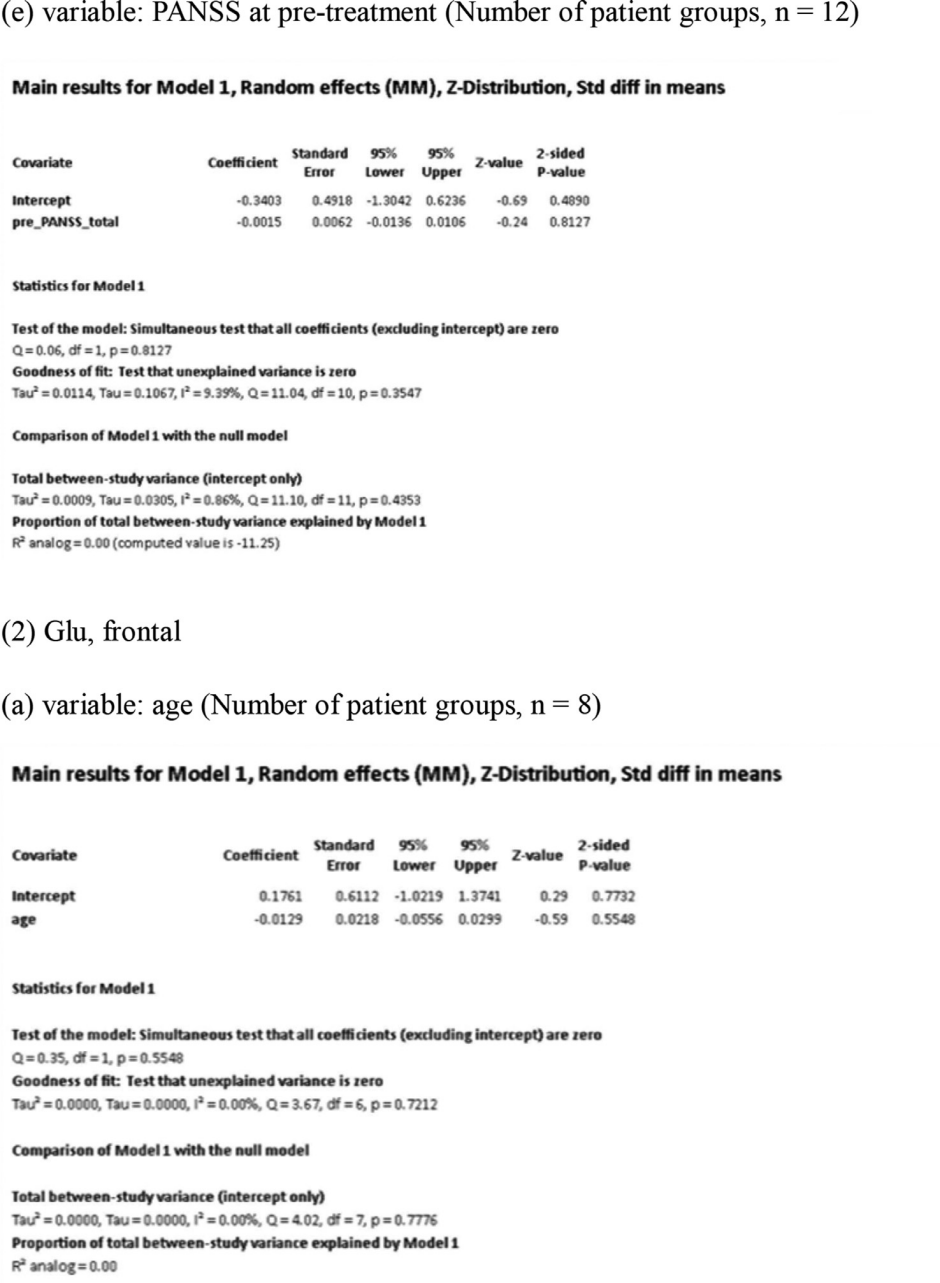

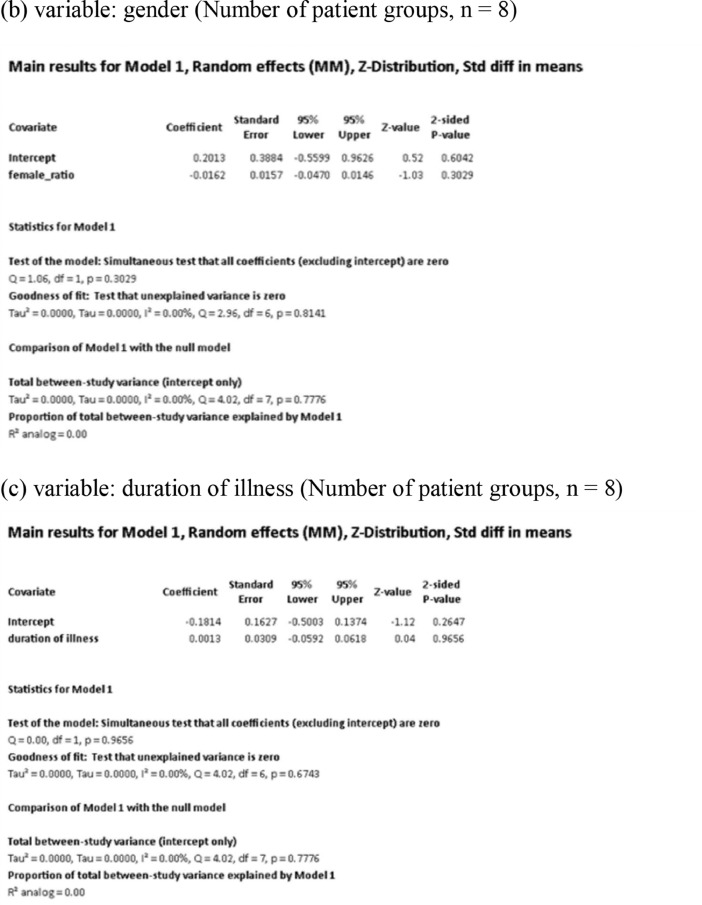

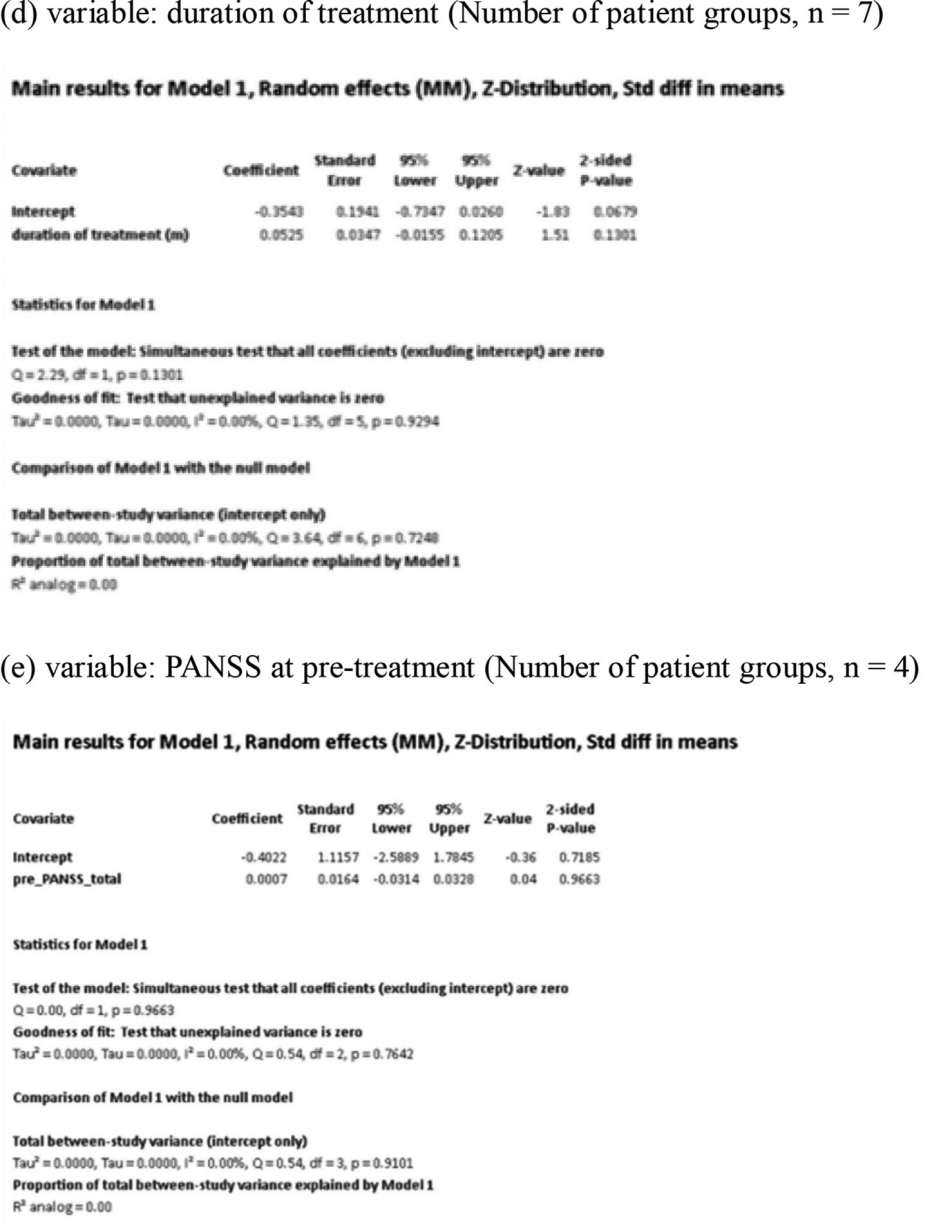

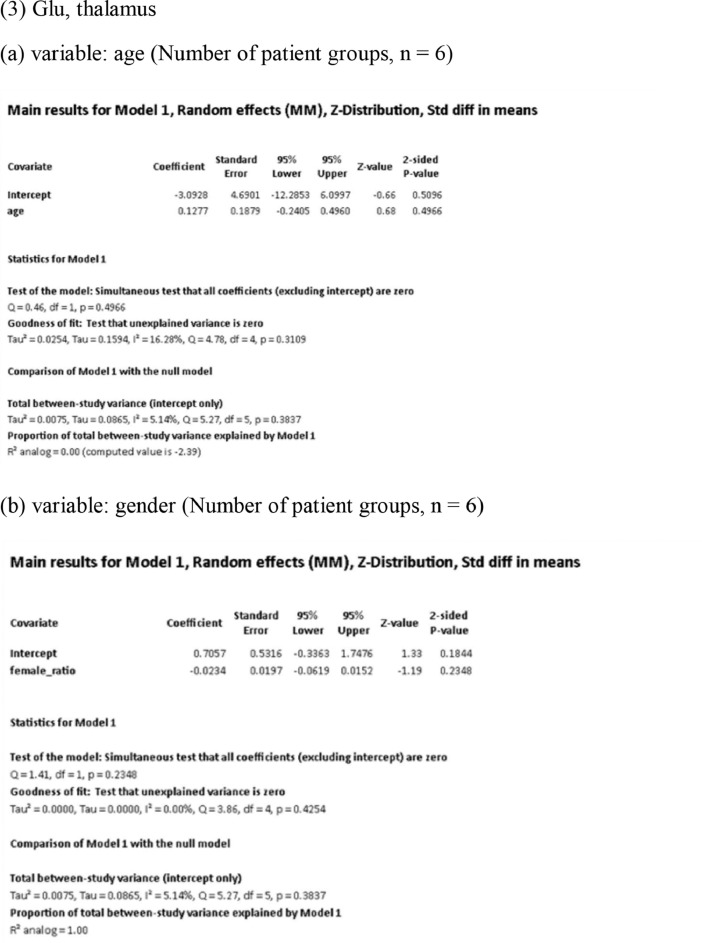

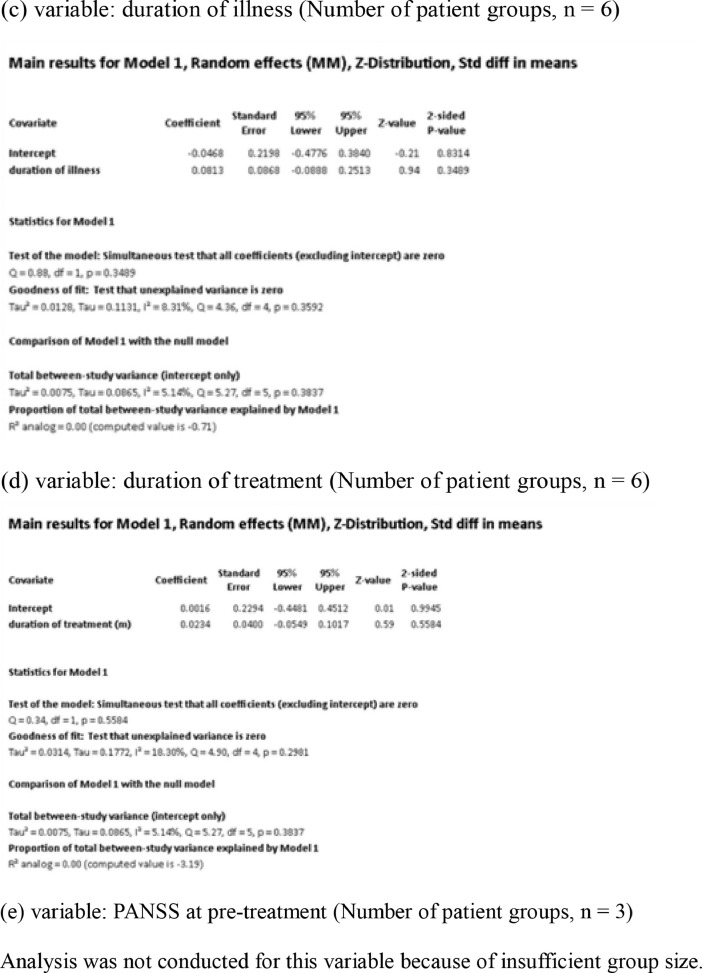

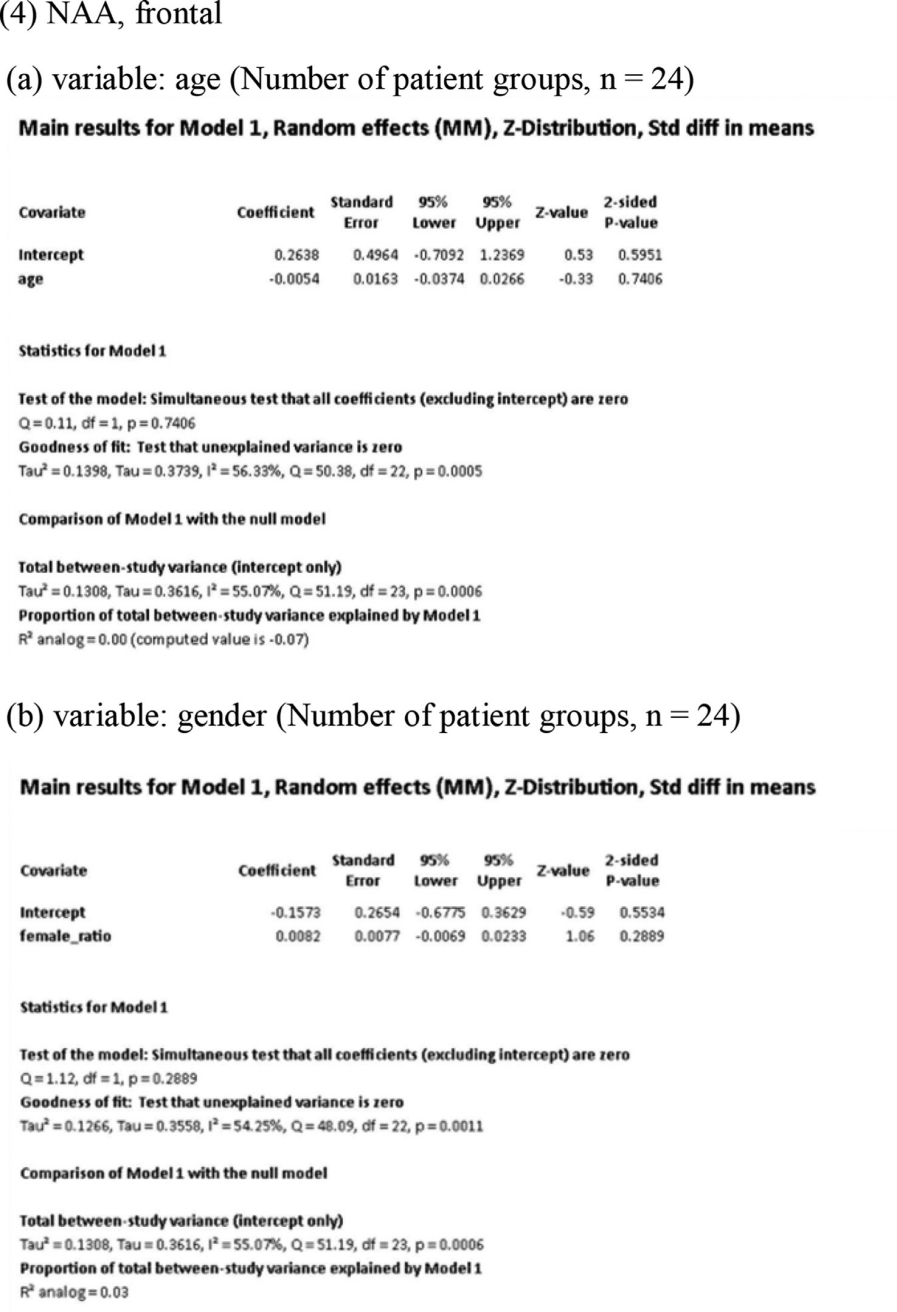

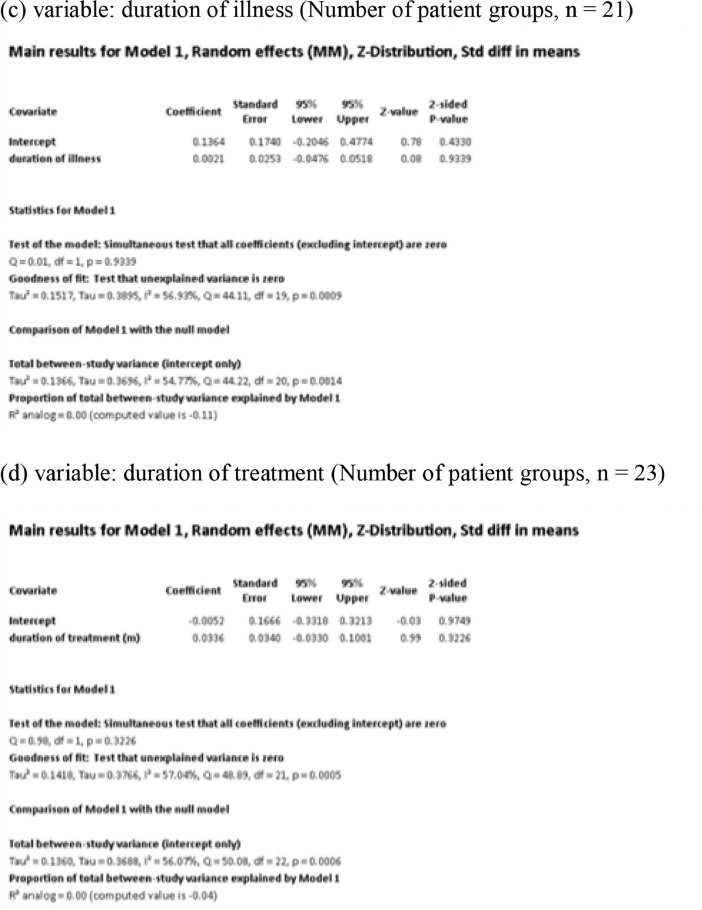

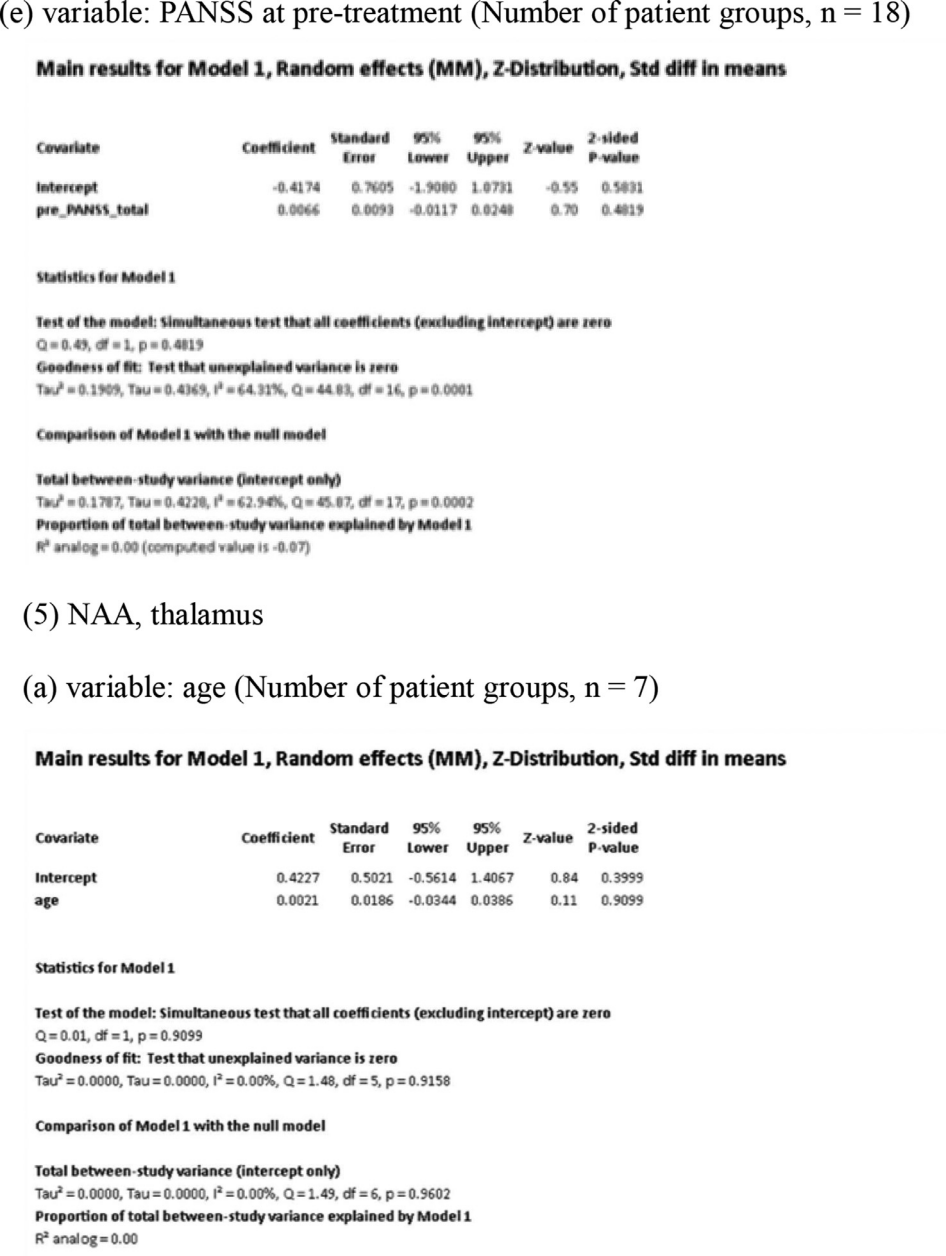

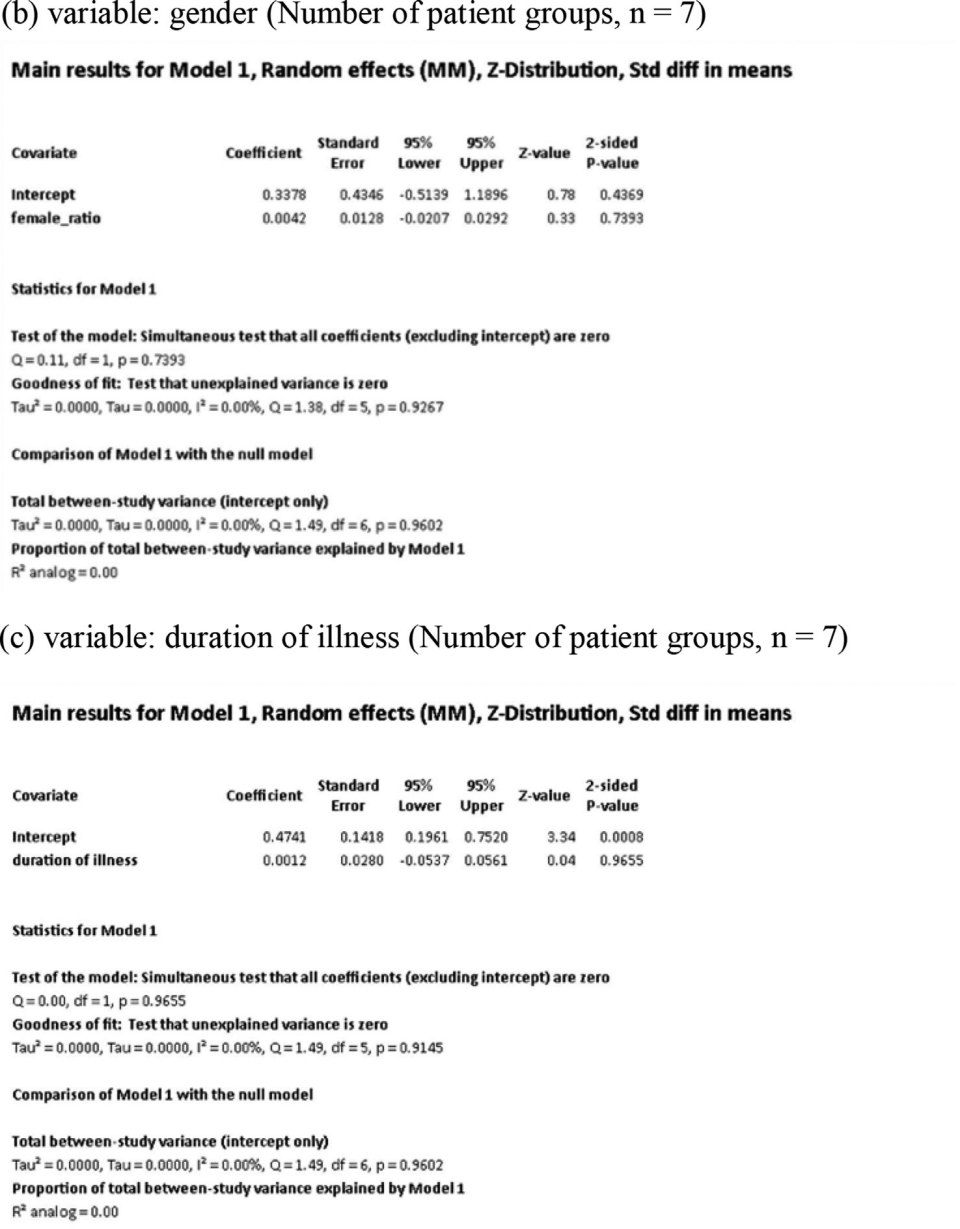

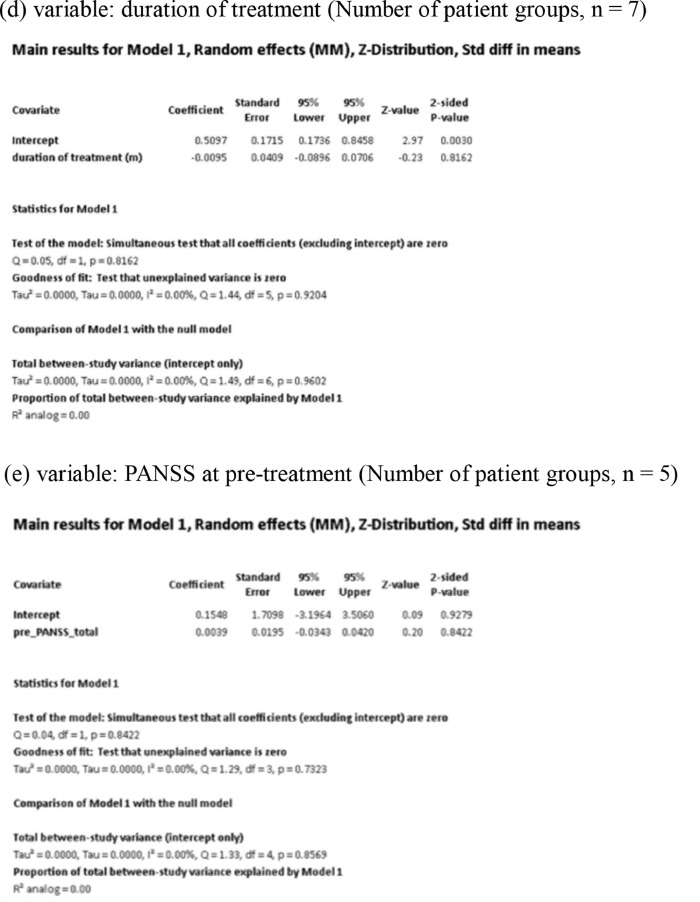

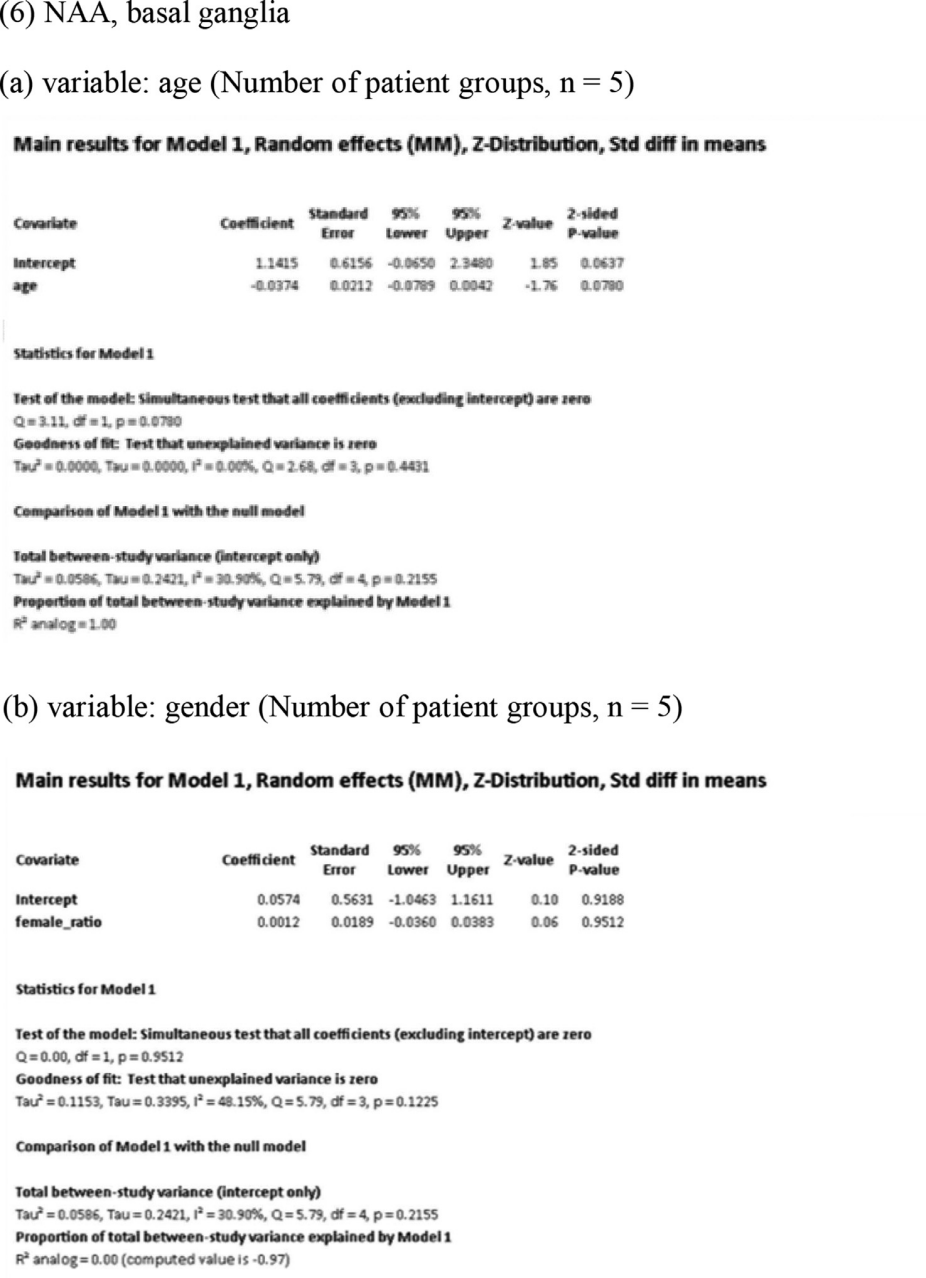

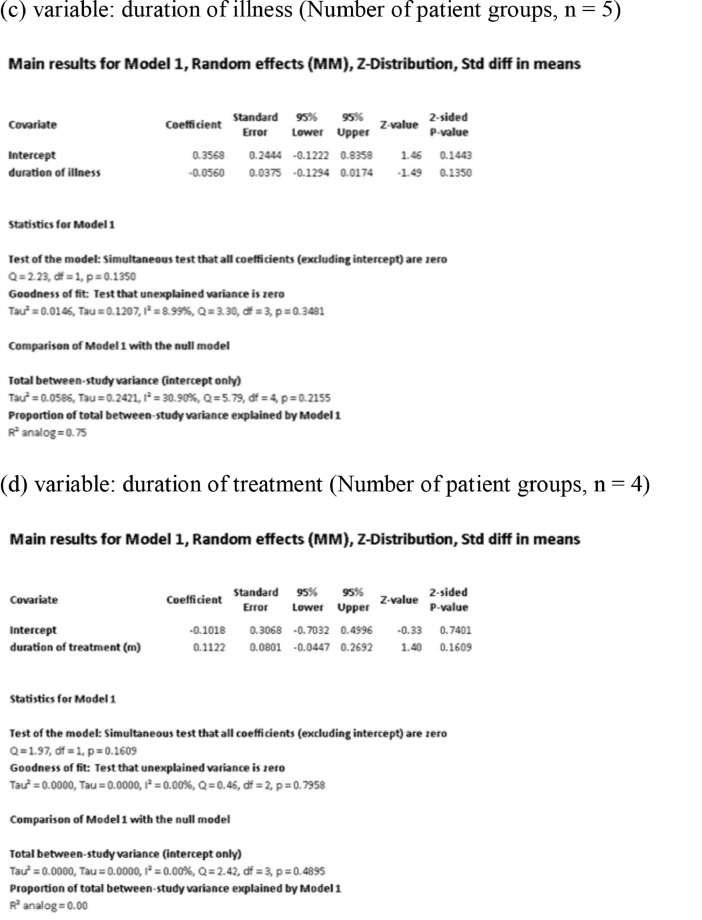

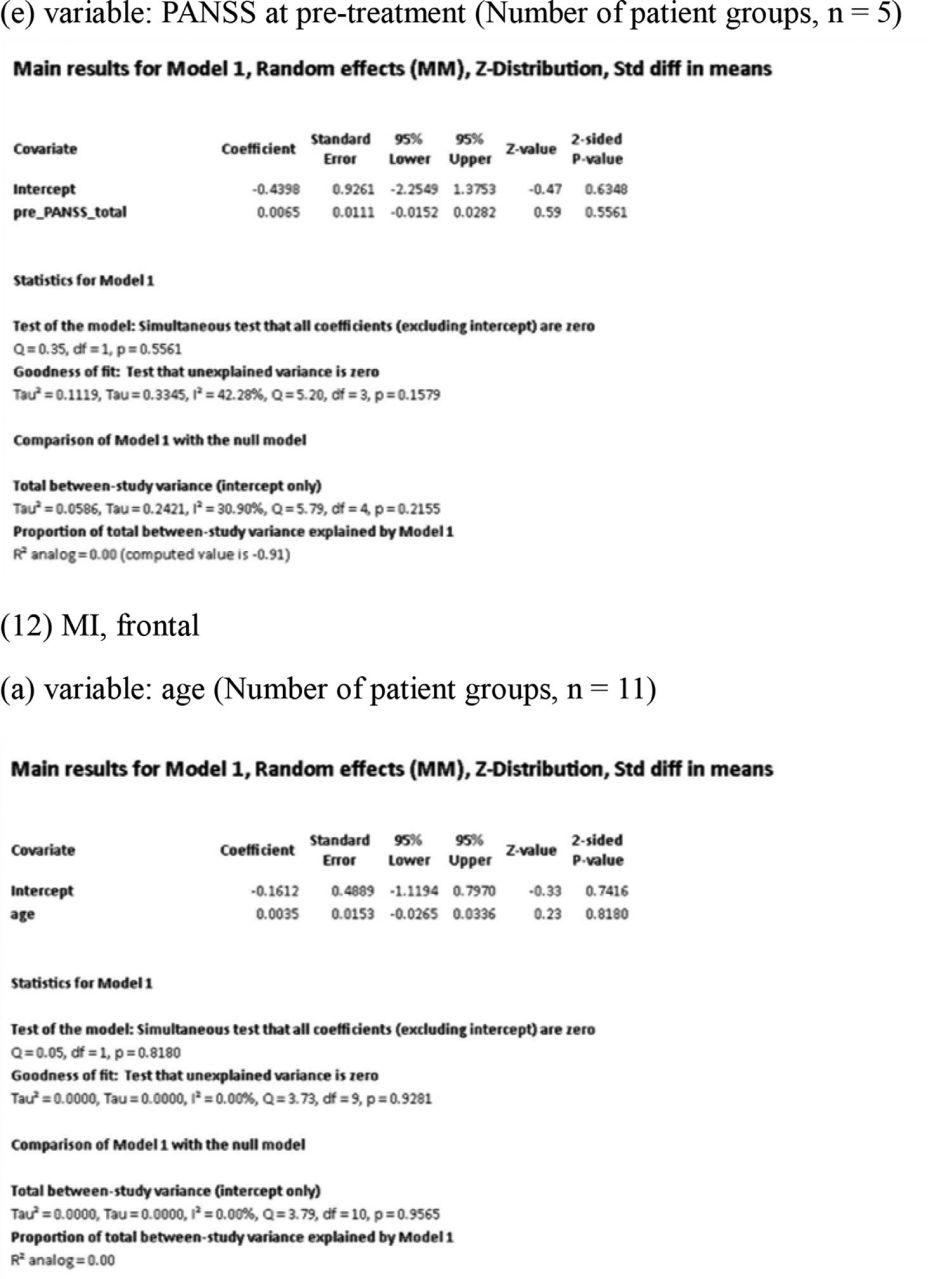

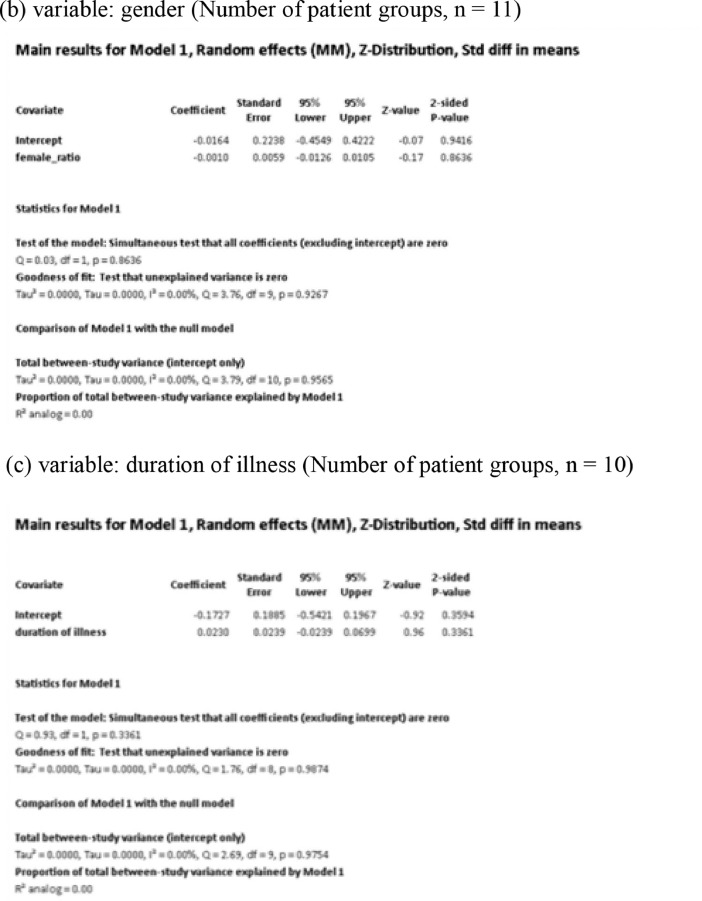

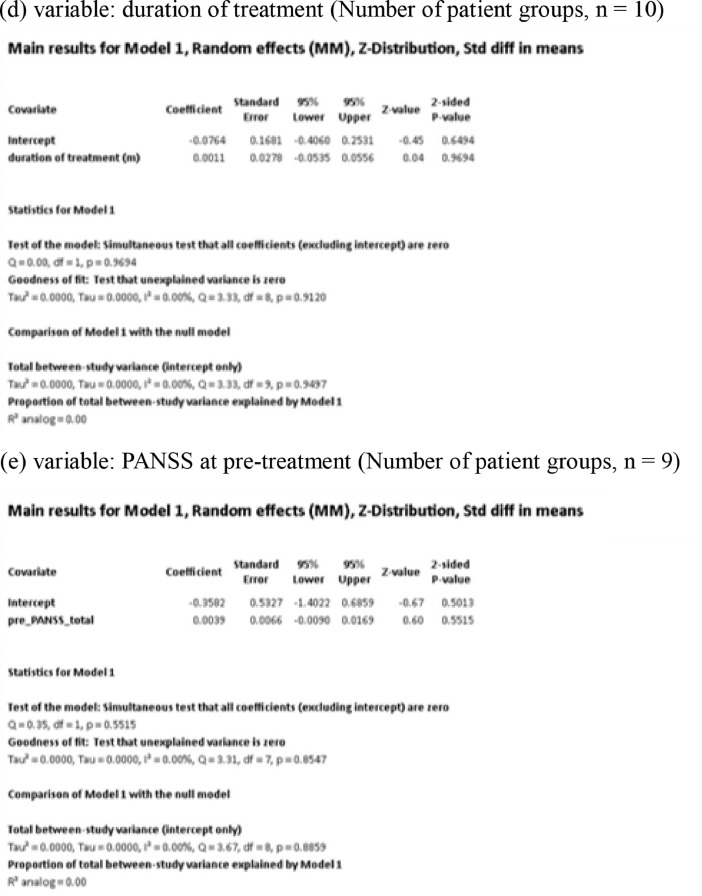
Meta-regression analyses for investigating the effects of clinico-demographic variables on neurometabolites. The analyses were conducted for regions of interest (ROIs) of five patient groups or more. Abbreviations: Glu, glutamate; Gln, glutamine; Glx, glutamate + glutamine; NAA, *N*-acetylaspartate; MI, myo-inositol; PANSS, Positive and Negative Syndrome Scale.

Fig. 1 demonstrates meta-regression analyses to investigate the effects of clinico-demographic variables on neurometabolites.

**Fig. 2:**
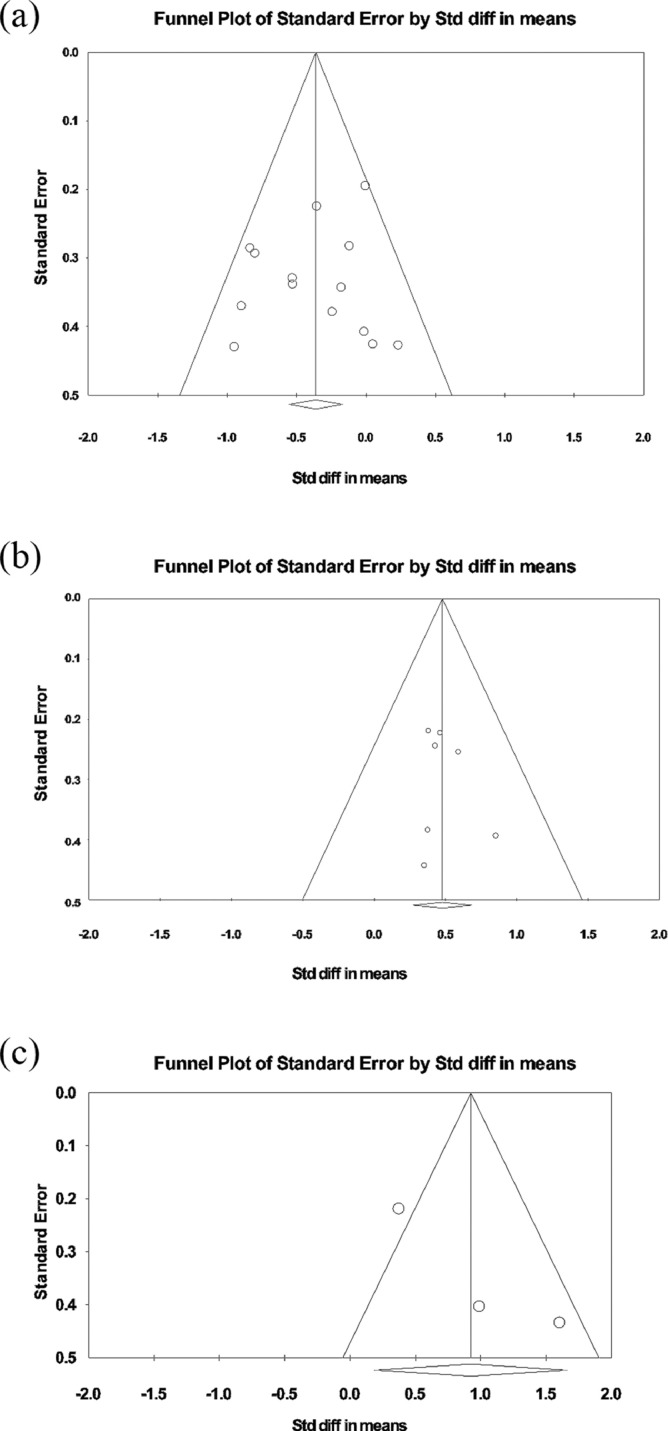
Funnel plot of (a) glutamate + glutamine (Glx) differences in the frontal cortex, (b) *N*-acetylaspartate (NAA) differences in the thalamus, and (c) myo-inositol (MI) differences in the thalamus.

Fig. 2 depicts a funnel plot of frontal Glx, thalamic NAA, and thalamic MI.

Datasheet 1 includes the comprehensive data with a summary sheet for our meta-analysis.

## 2 Experimental design, materials, and methods

### 2.1 Data search

#### 2.1.1 Literature search

Two authors (MK and SM) initially screened the titles and abstracts of articles to identify potentially relevant data. These authors then assessed the eligibility of these data for our meta-analysis, which required full-text screening. Discrepancies in data selection were resolved by discussions. We excluded articles in which only figures for MRS data were reported and metabolite values were not available despite our inquiry.

We performed the literature search on February 6, 2019 with MEDLINE (1946 to January week 4, 2019), Embase (1947 to February 05, 2019), and PsycINFO (1806 to January week 4, 2019). Final search was performed on March 01, 2019, and further data were retrieved for inclusion.

#### 2.1.2 Data extraction

Two authors, MK and SM, independently extracted data. The data were then cross-checked and discrepancies were resolved by discussion between these two authors. When different articles reported data on the same metabolite from the same sample, we chose the article with the larger sample size for inclusion in our meta-analysis. The extracted data included the name of the first author, year of publication, number of patients at pre- and post-treatment, mean age of patients at pre-treatment, sex ratio of patients, clinical characteristics and symptom severity of patients, baseline treatment, duration of illness, detailed type of treatment, duration of treatment, strength of magnetic field, MRS acquisition sequence, echo time, repetition time, value of neurometabolites (mean, sd, number) at pre- and post-treatment, and scaling (creatine or water scaling, corrected by cerebrospinal fluid or not).

#### 2.1.3 Eligibility criteria

Eligibility criteria were as follows:

1) Data for patients meeting the Diagnostic and Statistical Manual of Mental Disorders, 3rd, 4th, or 5th edition criteria for psychotic disorders including schizophrenia, schizoaffective, and schizophreniform; or patients meeting the Comprehensive Assessment of At-Risk Mental States criteria for being at ultra-high risk (UHR) for onset of first psychotic disorder
2) Data with neurometabolite levels (Glu, Gln, Glx, GABA, NAA or MI) for both pre- and post-treatment using ^1^H-MRS
3) Data with at least five patients at each time point
4) Data sufficient to obtain mean differences between two time points
5) Data from English-language articles

Exclusion criteria were as follows:

1) Cross-sectional data (only one time point)
2) Data without sufficient information for the meta-analysis regardless of our inquiry from authors
3) Non-English articles and conference abstracts

5017 articles were identified through our initial database search. Among them, 32 articles met the eligibility criteria for our meta-analysis. From these article records, we retrieved data for 39 patient groups.

## 3 Outcomes

We investigated changes in neurometabolite levels between pre- and post-treatment in the following ROIs:

1) frontal cortex including frontal white matter, anterior cingulate cortex, medial prefrontal cortex, and dorsolateral prefrontal cortex
2) temporal cortex
3) parieto-occipital cortex
4) thalamus
5) basal ganglia
6) hippocampus

MRS data in the cerebellum were not investigated because of a lack of sufficient data size.

When bilateral data were reported, only those of the left hemisphere were included, as it was examined in most research. In case the metabolite data of the same sample were reported from two different sub-regions within the same ROI, we included data for the sub-region more frequently used by others. If an article reported two or more kinds of measures of metabolites, we prioritized an absolute metabolite value with cerebrospinal fluid ratio correction, and if not available, we used the ratio of a metabolite to the creatine level. If data of three or more time points were reported from the same publication, we used data at the first follow-up as well as at baseline to minimize other effects on metabolites.

## 4 Meta-analysis

1) Software used for meta-analysis: Review Manager Version 5.3 (http://tech.cochrane.org/revman)
2) Statistical methods: random-effects model, standardized mean difference (SMD) method between pre- and post-treatment
3) Index of heterogeneity: Q-test and I^2^ index

Characteristics of the included data of the meta-analysis are shown in Datasheet 1.

## 5 Moderator analyses

### 5.1 Subgroup analyses

#### 5.1.1 Effects of treatment type

Because type of treatment could influence changes in neurometabolite status, we performed the meta-analysis by dividing the patient groups into two subgroups: AP subgroups and non-AP subgroups.

#### 5.1.2 Effects of ROI location within the frontal cortex

Because previous MRS research indicated that the location of ROIs within the frontal cortex might affect the neurometabolite status, we divided the frontal ROIs based on 1) whether they were principally composed of gray matter or white matter, and 2) whether they were located in the ventral part or dorsal part of the frontal lobe, and investigated neurometabolite changes in these frontal sub-regions separately.

Subgroup analyses were conducted if they were based on three or more patient groups.

### 5.2 Meta-regression analyses

Using Comprehensive Meta-Analysis version 3, we performed meta-regression analyses to investigate the effects of clinico-demographic variables on neurometabolites. The analyses were conducted for ROIs of five patient groups or more. The variables investigated include age, gender, illness duration, treatment duration, and symptom severity at baseline measured by Positive and Negative Syndrome Scale [3] (Fig. 1).

## 6 Publication bias

For regions and metabolites in which significant treatment effect was found, we investigated publication bias by visual inspection of a funnel plot and by using Begg and Mazumdar rank correlation, with Comprehensive Meta-Analysis version 3. We did not find any publication bias for frontal cortex Glx, thalamus NAA and thalamic MI (tau = 0.15, *p* = 0.49; tau = −0.07, *p* = 0.85; tau = 1.00, *p* = 0.11, respectively) (Fig. 2).

## 7 Quality assessment

Risk of bias was assessed by modified Newcastle - Ottawa Quality Assessment Scale [4]. Participants' selection (case definition, representativeness, ascertainment of exposure, definition of controls) and exposure (outcome assessment, follow-up period, adequacy of follow-up) were independently scored by two review authors, MK and SM. Discrepancies were resolved by discussion between the two. Higher scores indicate better quality (maximum total score = 14).

## Declaration of Competing Interest

The authors declare that they have no known competing financial interests or personal relationships which have, or could be perceived to have influenced the work reported in this article.

## Appendix Supplementary materials

Image, application 1
